# Received signal strength and local terrain profile data for radio network planning and optimization at GSM frequency bands

**DOI:** 10.1016/j.dib.2017.12.036

**Published:** 2017-12-19

**Authors:** Segun I. Popoola, Aderemi A. Atayero, Nasir Faruk

**Affiliations:** a Department of Electrical and Information Engineering, Covenant University, Ota, Nigeria; b Department of Telecommunication Science, University of Ilorin, Ilorin, Nigeria

**Keywords:** Received signal strength, GSM, Path loss, Radio network planning, Radio network optimization, Smart and connected communities

## Abstract

The behaviour of radio wave signals in a wireless channel depends on the local terrain profile of the propagation environments. In view of this, Received Signal Strength (RSS) of transmitted signals are measured at different points in space for radio network planning and optimization. However, these important data are often not publicly available for wireless channel characterization and propagation model development. In this data article, RSS data of a commercial base station operating at 900 and 1800 MHz were measured along three different routes of Lagos-Badagry Highway, Nigeria. In addition, local terrain profile data of the study area (terrain elevation, clutter height, altitude, and the distance of the mobile station from the base station) are extracted from Digital Terrain Map (DTM) to account for the unique environmental features. Statistical analyses and probability distributions of the RSS data are presented in tables and graphs. Furthermore, the degree of correlations (and the corresponding significance) between the RSS and the local terrain parameters were computed and analyzed for proper interpretations. The data provided in this article will help radio network engineers to: predict signal path loss; estimate radio coverage; efficiently reuse limited frequencies; avoid interferences; optimize handover; and adjust transmitted power level.

**Specifications Table**

**Table:** 

Subject area	*Engineering*
More specific subject area	*Communication Engineering*
Type of data	*Tables, graphs, figures, and spreadsheet file*
How data was acquired	*An extensive drive test measurement was conducted and RSS data were collected over a commercial base station at 900 and 1800* *MHz using Ericsson TEMS 9.1.1 investigation software running on a Windows 7 operating system laptop, W995 TEMS mobile phones, and Garmin Global Positioning System (GPS). Local terrain profile information were obtained from digital terrain map loaded on ATOLL radio network planning tool.*
Data format	*Raw, analyzed*
Experimental factors	*The base station used during RSS measurement was a commercial equipment. The specifications and network parameters were strictly adhered to as suggested by the manufacturer.*
Experimental features	*Statistical analyses and probability distributions of the RSS data are presented. Correlation analyses of the RSS data and local terrain profile information were performed to understand the relationships between the two datasets.*
Data source location	*The received signal strength and local terrain profile information provided in this article were collected along Lagos-Badagry Highway, Lagos State, Nigeria (Latitude 6.47385*^*o*^*N, Longitude 3.00408*^*o*^*E)*
Data accessibility	*A comprehensive datasets on received signal strength and local terrain profile at GSM frequency bands (900 MHz and 1800* *MHz) are provided in this article in easily reusable format.*

**Value of the data**

• The data provided in this article will facilitate research advancement in wireless channel characterization that accounts for local terrain features of the Nigerian propagation environment [1], [2], [3], [4], [5].
• RSS data and local terrain profile information are useful for the design, formulation, and development of radio propagation models. These models are used to predict the mean received signal strength of a wireless network at a given distance of the receiver away from the transmitter. Accurate and efficient path loss models are highly essential for signal power predictions at different points within the coverage area during radio network planning and optimization [6], [7], [8], [9].
• Radio network engineers rely on RSS data and local terrain profile information to: determine optimal locations of base stations; achieve best possible data rates; estimate radio coverage; determine the required transmission power; aid appropriate selection of antenna height and pattern; perform efficient frequency allocation; conduct radio network optimization; perform interference feasibility studies; and ensure an acceptable level of quality of service without the need of expensive and time consuming measurements [10], [11], [12], [13], [14].

## 1 Data

Mobile networks provide the infrastructure for global interconnectedness and digital inclusion that are required to achieve the set targets of the 2030 Sustainable Development Goals (SDGs) agenda. There is a continuous exponential increase in the number of wireless devices, mobile applications, and services that require connectivity to cellular networks in different scenarios and use cases. With the emerging trends in Internet of Things (IoT) and Machine-to-Machine (M2M) communication technologies, more than 100 billion smart devices and sensors are expected to be connected by 2020 [15]. However, the capacity of the state-of-the-art cellular networks may not be sufficient enough to meet the high user requirements of future wireless communications. One of the easiest way of increasing cellular network capacity is to deploy more base stations in a given coverage area. More base stations will be deployed in the future to guarantee quality signal reception at every point within the coverage area. A correct knowledge of electromagnetic wave propagation is required for efficient radio network planning and optimization [8], [9], [10], [11], [12].

**Fig. 1:**
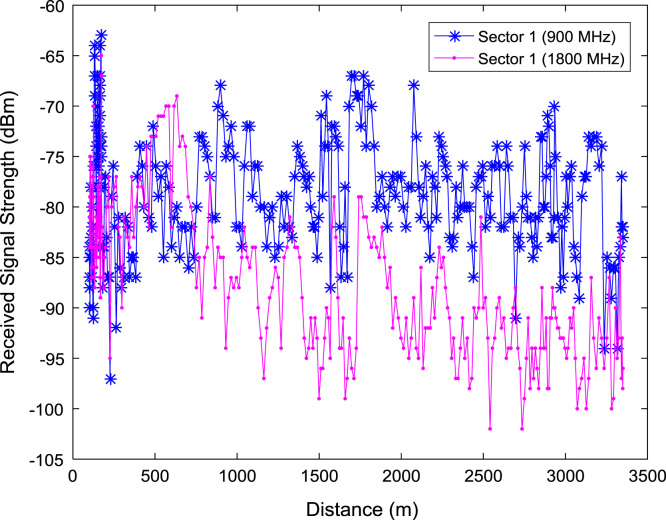
RSS variations with distance along drive route 1.

**Fig. 2:**
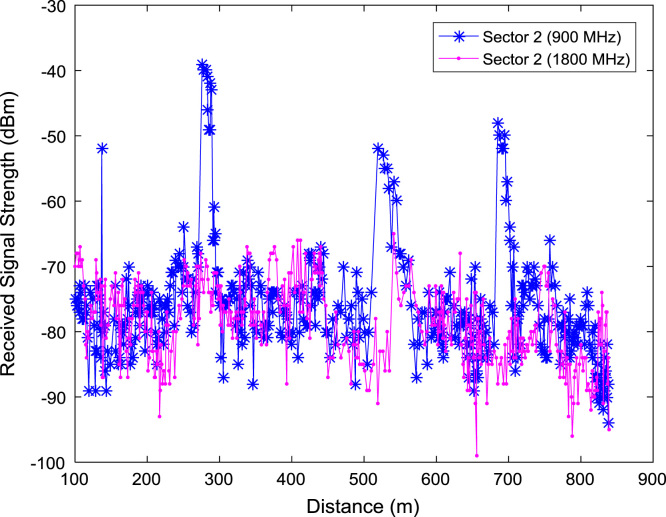
RSS variations with distance along drive route 2.

**Fig. 3:**
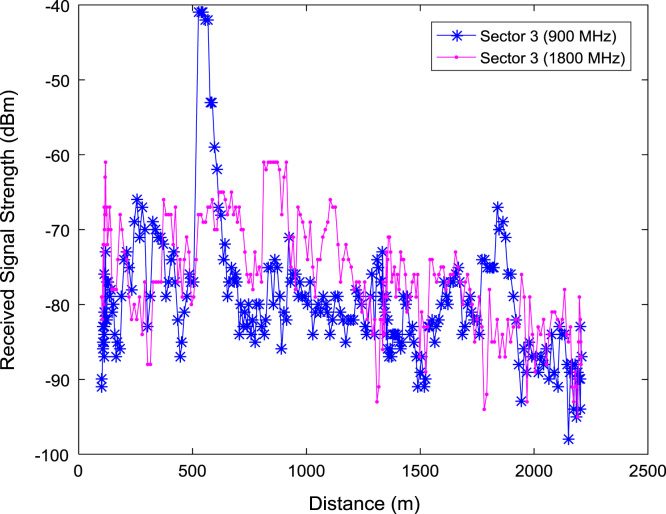
RSS variations with distance along drive route 3.

**Fig. 4:**
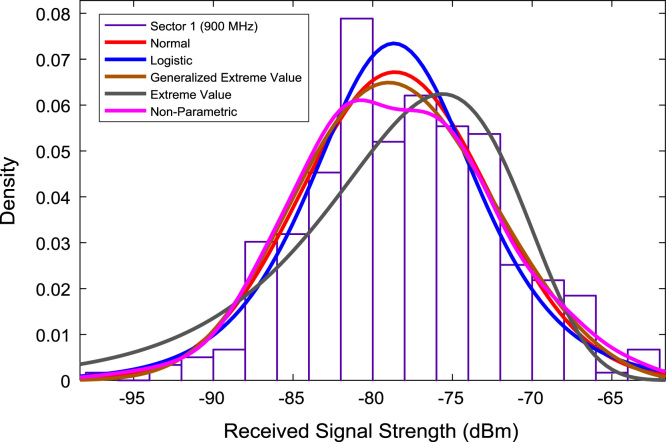
Probability distribution of RSS data along sector 1 (900 MHz).

**Fig. 5:**
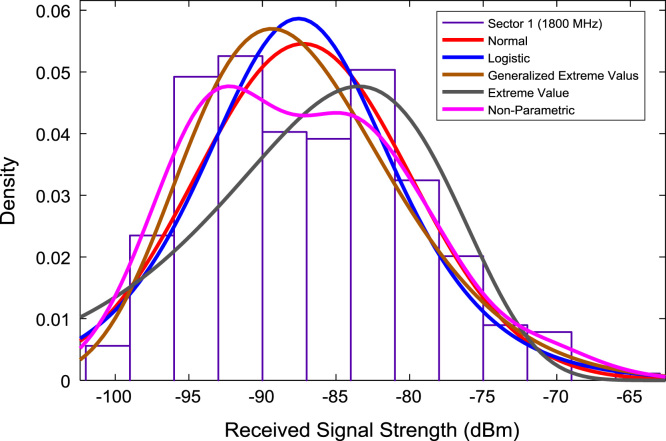
Probability distribution of RSS data along sector 1 (1800 MHz).

**Fig. 6:**
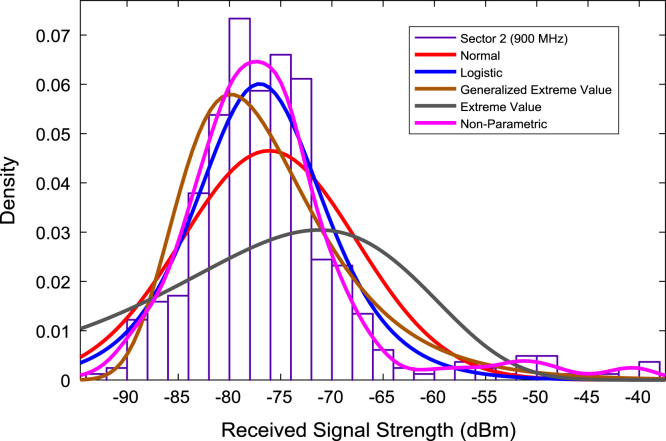
Probability distribution of RSS data along sector 2 (900 MHz).

**Fig. 7:**
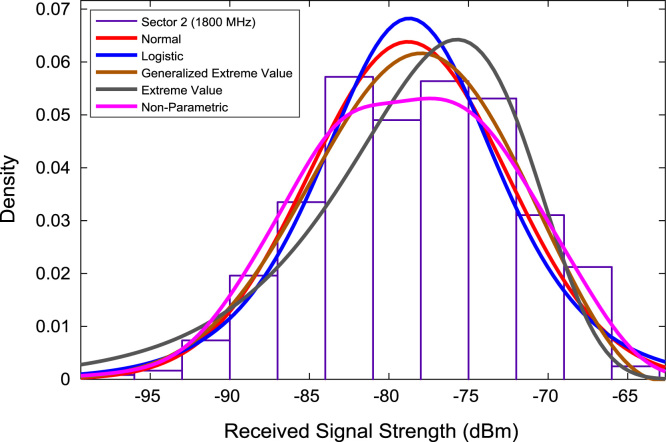
Probability distribution of RSS data along sector 2 (1800 MHz).

**Fig. 8:**
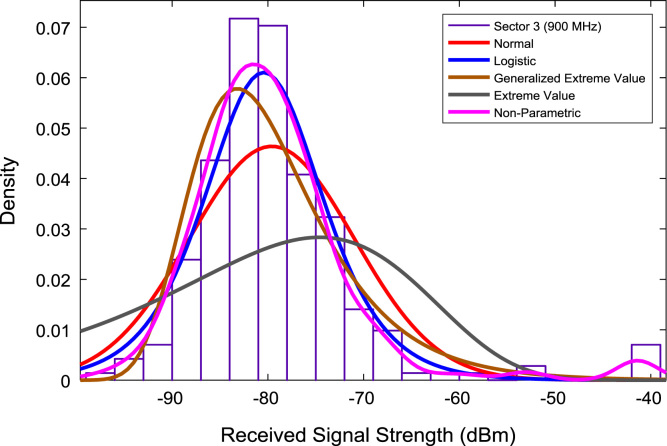
Probability distribution of RSS data along sector 3 (900 MHz).

**Fig. 9:**
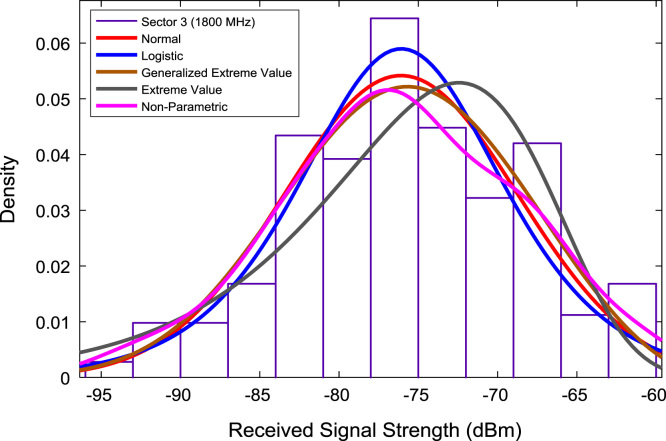
Probability distribution of RSS data along sector 3 (1800 MHz).

In this data article, RSS data of a commercial base station operating at 900 and 1800 MHz were measured along three different routes of Lagos-Badagry Highway, Nigeria. Statistical analyses of the RSS data along the three sectors of the base station are presented in Table 1. Fig. 1, Fig. 2, Fig. 3 show the RSS variations with distance along drive routes 1, 2, and 3. Fig. 4, Fig. 5, Fig. 6, Fig. 7, Fig. 8, Fig. 9 show the probability distribution of RSS Data along the three sectors and across Global System for Mobile communications (GSM) frequencies (900 and 1800 MHz).

**Table 1:** Descriptive first-order statistics of RSS data.

	***Sector 1*****(900 MHz)**	***Sector 1* (1800 MHz)**	***Sector 2*****(900 MHz)**	***Sector 2*****(1800 MHz)**	***Sector 3*****(900 MHz)**	***Sector 3*****(1800 MHz)**
Mean	−78.61	−87.23	−76.13	−78.78	−79.59	−76.09
Median	−79.00	−88.00	−77.00	−79.00	−81.00	−76.00
Mode	−82.00	−94.00	−79.00	−77.00	−79.00	−77.00
Standard Deviation	5.94	7.31	8.58	6.26	8.60	7.36
Variance	35.25	53.44	73.53	39.16	73.97	54.23
Kurtosis	2.92	2.66	7.57	2.51	9.90	2.68
Skewness	0.05	0.42	1.73	−0.15	2.03	−0.09
Range	34.00	37.00	55.00	34.00	57.00	34.00
Minimum	−97.00	−102.00	−94.00	−99.00	−98.00	−95.00
Maximum	−63.00	−65.00	−39.00	−65.00	−41.00	−61.00

## 2 Experimental design, materials and methods

**Fig. 10:**
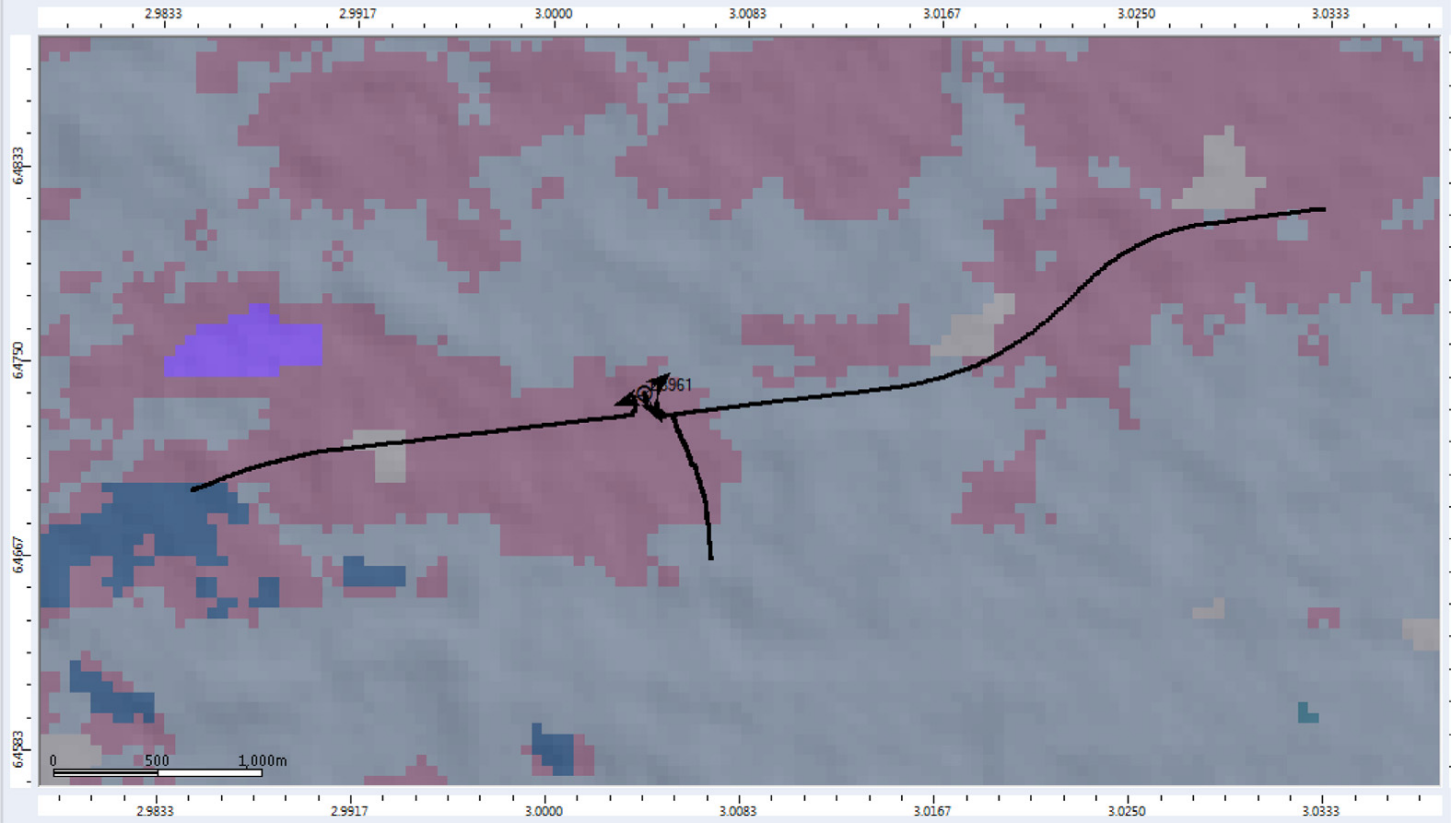
Drive test location on Digital Terrain Map (DTM).

An extensive measurement campaign was conducted along Lagos-Badagry Expressway, Nigeria. The study area is located at 6°29'15.22" N, 3°08'07.15" E. The propagation environment is a typical rural area and it is composed of scattered buildings, foliage, and open lands. Three different routes were mapped out, as shown in Fig. 10, to span the radio coverage area of a base transceiver station operating at 900 and 1800 MHz. The RF measurements were carried out under good climatic conditions. Also, good vehicular accessibility to site locations were considered for a smooth test drive. Distances covered by the drive routes were considered long enough to allow the noise floor of the receiver to be reached. The data collection process was performed with the use of the Transmission Evaluation and Monitoring System (TEMS) network performance investigation software. TEMS Investigation has data collection, real-time network data analysis, and post-data processing capabilities. This network testing software ran on an Intel Core i5-3210MCPU@2.50 GHz speed with 4 GB RAM and 64-bit Windows 7 operating system. A TEMS mobile station, the software USB dongle, and a Garmin Global Positioning System (GPS) were connected to the laptop. The whole set-up was carefully placed in a vehicle, and the vehicle was driven at an average speed of 40 km/h. This speed was maintained to minimize Doppler effects.

Table 2, Table 3, Table 4, Table 5, Table 6, Table 7, Table 8, Table 9, Table 10, Table 11, Table 12, Table 13 present the matrix of correlation coefficients and the matrix of *p*-values for testing the hypothesis that there is no relationship between the observed phenomena (null hypothesis). If an off-diagonal element of *P* is smaller than the significance level of 0.05, then the corresponding correlation is considered significant.

**Table 2:** Matrix of correlation coefficients for data on sector 1 (900 MHz).

	**Terrain elevation**	**Clutter height**	**Altitude**	**Distance**	**RSS**
**Terrain elevation**	1.0000				
**Clutter height**	0.2959	1.0000			
**Altitude**	0.2125	0.2104	1.0000		
**Distance**	−0.0244	−0.2046	0.6733	1.0000	
**RSS**	0.1543	0.0115	−0.2548	−0.1297	1.0000

**Table 3:** Matrix of *P*-values for data on sector 1 (900 MHz).

	**Terrain elevation**	**Clutter height**	**Altitude**	**Distance**	**RSS**
**Terrain elevation**	1.0000				
**Clutter height**	0.0000	1.0000			
**Altitude**	0.0002	0.0003	1.0000		
**Distance**	0.6748	0.0004	0.0000	1.0000	
**RSS**	0.0076	0.8428	0.0000	0.0252	1.0000

**Table 4:** Matrix of correlation coefficients for data on sector 1 (1800 MHz).

	**Terrain elevation**	**Clutter height**	**Altitude**	**Distance**	**RSS**
**Terrain elevation**	1.0000				
**Clutter height**	0.2992	1.0000			
**Altitude**	0.2121	0.2140	1.0000		
**Distance**	−0.0241	−0.1975	0.6733	1.0000	
**RSS**	0.0584	0.1646	−0.4542	−0.7152	1.0000

**Table 5:** Matrix of *P*-values for data on sector 1 (1800 MHz).

	**Terrain elevation**	**Clutter height**	**Altitude**	**Distance**	**RSS**
**Terrain celevation**	1.0000				
**Clutter height**	0.0000	1.0000			
**Altitude**	0.0002	0.0002	1.0000		
**Distance**	0.6792	0.0006	0.0000	1.0000	
**RSS**	0.3151	0.0044	0.0000	0.0000	1.0000

**Table 6:** Matrix of correlation coefficients for data on sector 2 (900 MHz).

	**Terrain elevation**	**Clutter height**	**Altitude**	**Distance**	**RSS**
**Terrain elevation**	1.0000				
**Clutter height**	0.7931	1.0000			
**Altitude**	−0.4598	−0.3770	1.0000		
**Distance**	0.6284	0.7350	0.1410	1.0000	
**RSS**	−0.2830	−0.1120	0.1589	−0.1803	1.0000

**Table 7:** Matrix of *P*-values for data on sector 2 (900 MHz).

	**Terrain elevation**	**Clutter height**	**Altitude**	**Distance**	**RSS**
**Terrain elevation**	1.0000				
**Clutter height**	0.0000	1.0000			
**Altitude**	0.0000	0.0000	1.0000		
**Distance**	0.0000	0.0000	0.0043	1.0000	
**RSS**	0.0000	0.0235	0.0013	0.0002	1.0000

**Table 8:** Matrix of correlation coefficients for data on sector 2 (1800 MHz).

	**Terrain elevation**	**Clutter height**	**Altitude**	**Distance**	**RSS**
**Terrain elevation**	1.0000				
**Clutter height**	0.7943	1.0000			
**Altitude**	−0.4563	−0.3766	1.0000		
**Distance**	0.6250	0.7283	0.1499	1.0000	
**RSS**	−0.3900	−0.3701	0.0444	−0.4063	1.0000

**Table 9:** Matrix of *P*-values for data on sector 2 (1800 MHz).

	**Terrain elevation**	**Clutter height**	**Altitude**	**Distance**	**RSS**
**Terrain elevation**	1.0000				
**Clutter height**	0.0000	1.0000			
**Altitude**	0.0000	0.0000	1.0000		
**Distance**	0.0000	0.0000	0.0024	1.0000	
**RSS**	0.0000	0.0000	0.3711	0.0000	1.0000

**Table 10:** Matrix of correlation coefficients for data on sector 3 (900 MHz).

	**Terrain elevation**	**Clutter height**	**Altitude**	**Distance**	**RSS**
**Terrain elevation**	1.0000				
**Clutter height**	0.3586	1.0000			
**Altitude**	0.6586	0.4438	1.0000		
**Distance**	0.6537	0.2213	0.6512	1.0000	
**RSS**	−0.3060	−0.1509	−0.1946	−0.3811	1.0000

**Table 11:** Matrix of *P*-values for data on sector 3 (900 MHz).

	**Terrain elevation**	**Clutter height**	**Altitude**	**Distance**	**RSS**
**Terrain elevation**	1.0000				
**Clutter height**	0.0000	1.0000			
**Altitude**	0.0000	0.0000	1.0000		
**Distance**	0.0000	0.0006	0.0000	1.0000	
**RSS**	0.0000	0.0201	0.0026	0.0000	1.0000

**Table 12:** Matrix of correlation coefficients for data on sector 3 (1800 MHz).

	**Terrain elevation**	**Clutter height**	**Altitude**	**Distance**	**RSS**
**Terrain elevation**	1.0000				
**Clutter height**	0.3586	1.0000			
**Altitude**	0.6568	0.4418	1.0000		
**Distance**	0.6528	0.2227	0.6535	1.0000	
**RSS**	−0.3517	−0.1911	−0.1768	−0.5799	1.0000

**Table 13:** Matrix of *P*-values for data on sector 3 (1800 MHz).

	**Terrain elevation**	**Clutter height**	**Altitude**	**Distance**	**RSS**
**Terrain elevation**	1.0000				
**Clutter height**	0.0000	1.0000			
**Altitude**	0.0000	0.0000	1.0000		
**Distance**	0.0000	0.0005	0.0000	1.0000	
**RSS**	0.0000	0.0031	0.0062	0.0000	1.0000
